# Contrasting Photophysiological Characteristics of Phytoplankton Assemblages in the Northern South China Sea

**DOI:** 10.1371/journal.pone.0153555

**Published:** 2016-05-19

**Authors:** Peng Jin, Guang Gao, Xin Liu, Futian Li, Shanying Tong, Jiancheng Ding, Zhihai Zhong, Nana Liu, Kunshan Gao

**Affiliations:** 1 State Key Laboratory of Marine Environmental Science, Xiamen University, Xiamen 361005, China; 2 Marine Biology Institute, Shantou University, Shantou, Guangdong 515063, China; CAS, CHINA

## Abstract

The growth of phytoplankton and thus marine primary productivity depend on photophysiological performance of phytoplankton cells that respond to changing environmental conditions. The South China Sea (SCS) is the largest marginal sea of the western Pacific and plays important roles in modulating regional climate and carbon budget. However, little has been documented on photophysiological characteristics of phytoplankton in the SCS. For the first time, we investigated photophysiological characteristics of phytoplankton assemblages in the northern South China Sea (NSCS) using a real-time *in*-*situ* active chlorophyll *a* fluorometry, covering 4.0 × 10^5^ km^2^. The functional absorption cross section of photosystem II (PSII) in darkness (σ_PSII_) or under ambient light (σ_PSII_’) (A^2^ quanta^-1^) increased from the surface to deeper waters at all the stations during the survey period (29 July to 23 August 2012). While the maximum (Fv/Fm, measured in darkness) or effective (Fq’/Fm’, measured under ambient light) photochemical efficiency of PSII appeared to increase with increasing depth at most stations, it showed inverse relationship with depth in river plume areas. The functional absorption cross section of PSII changes could be attributed to light-adapted genotypic feature due to niche-partition and the alteration of photochemical efficiency of PSII could be attributed to photo-acclimation. The chlorophyll *a* fluorometry can be taken as an analog to estimate primary productivity, since areas of higher photochemical efficiency of PSII coincided with those of higher primary productivity reported previously in the NSCS.

## 1. Introduction

Active chlorophyll *a* fluorometry has been now widely used in aquatic research [1–3] since it was firstly introduced to oceanography and limnology about 20 years ago [4]. Technological and commercial development has since packaged various fluorescence protocols into a number of platforms from submersible profilers to bench-top imagers then to the latest *in-situ* FIRe (Fluorescence Induction and Relaxation), which is a solution for real-time chlorophyll analysis, providing quick and continuous measurement. As a direct result, active chlorophyll *a* fluorometry has rapidly become an established tool by which scientists evaluate the response of aquatic primary producers to environmental changes.

One major use of active chlorophyll fluorescence in aquatic studies is to estimate primary productivity. In the northwest Atlantic Ocean, photosynthetic carbon fixation derived from chlorophyll fluorescence agreed well with that based on radiocarbon uptake, with a slope of 1.06 [4]. Similarly, this consistency has also been found in a field study in the Celtic Sea [5]. However, the relationship between chlorophyll fluorescence and carbon fixation can deviate when energy dissipation or transfer differ [6–8]. Furthermore, other electron sinks within the photosynthetic electron transfer chain (e.g. O_2_ uptake by the plastid terminal oxidase activity and/or the water-water cycle associated with the Mehler reaction) [9, 10] or those associated the Calvin Cycle (e.g., oxygenation of ribulose-1, 5-bisphosphate, RuBP) and nitrate assimilation [11], can lead to uncoupling of net O_2_ evolution or CO_2_ fixation from ETR in PSII. In addition, photorespiration is known to change the relationship [12]. Therefore, application of chlorophyll fluorescence to estimate primary productivity can be complicated due to photophysiological performances of the cells in different environments.

Another important use of active chlorophyll fluorescence technique is to determine the responses of aquatic primary producers to environmental changes. In subtropical and tropical Atlantic waters, maximum electron turnover rates (${ETR}_{RCII}^{max}$) correlated with mixed-layer depth and daily integrated photosynthetically active photon flux, whilst the absorption cross section of PSII inversely correlated with it due to the taxonomic and physiological differences in the phytoplankton communities [13]. In a shelf sea, absorption cross section of PSII showed dramatic variations as a result of changes in functional groups across the horizontal sessions while the maximum electron turnover and carbon fixation rates varied with depths as a result of photoacclimation [1]. Gao et al. have shown that variability of chlorophyll fluorescence could be caused by interactions of environmental factors [14]. The ratio of effective photochemical efficiency under high pCO_2_ to low pCO_2_ decreased from above 1 to below 1 with increased levels of light in diatoms [14]. The non-photochemical quenching of phytoplankton assemblages in the NSCS grown under high pCO_2_ was higher than that under low pCO_2_, which is more pronounced during noon period with high solar radiation [14]. Although photochemical parameters derived from fluorescence techniques are useful in analyzing phytoplankton species succession, community changes and primary productivity, documentation of these data in different regions is scarce and its applications to oceanographic studies are to be further explored.

The South China Sea (SCS), locating between the equator and 23.8°N, from 99.1 to 121.1°E, characterized by a tropical and subtropical climate, is the world’s largest marginal sea of the Pacific with a deep semi-closed basin and wide continental shelves. Numerous studies have demonstrated that photophysiological traits of phytoplankton (e.g., coordination and arrangement of the photosynthetic/photoprotective apparatus) have a marked impact on their growth and thus marine primary productivity in response to varying environmental conditions [1, 15, 16]. A recent modeling study by Liu et al. [17] underestimated the primary production in NSCS by 30% while excluding the photo-adaptation information of phytoplankton in the model. Therefore, it is crucial to investigate the photophysiological performance of natural phytoplankton assemblages under varying environmental regimes to improve the estimates of primary production.

To the best of our knowledge, research on *in-situ* photophysiological performances of phytoplankton assemblages in NSCS have yet to be undertaken. In the present study, the photophysiological performances of phytoplankton cells in NSCS were investigated during a cruise that covered 4.0 × 10^5^ km^2^ from 29 July to 23 August 2012 with the latest chlorophyll fluorescence technique, the *in situ* FIRe (Fluorescence Induction and Relaxation). We presented an insight on the photophysiological state of natural phytoplankton communities across a gradient of environmental variability, and showed that photochemical performances differ spatio-temporally with contrasting features found in river plume and upwelling areas.

## 2. Materials and Methods

### 2.1 Studied stations and sampling

**Fig 1:**
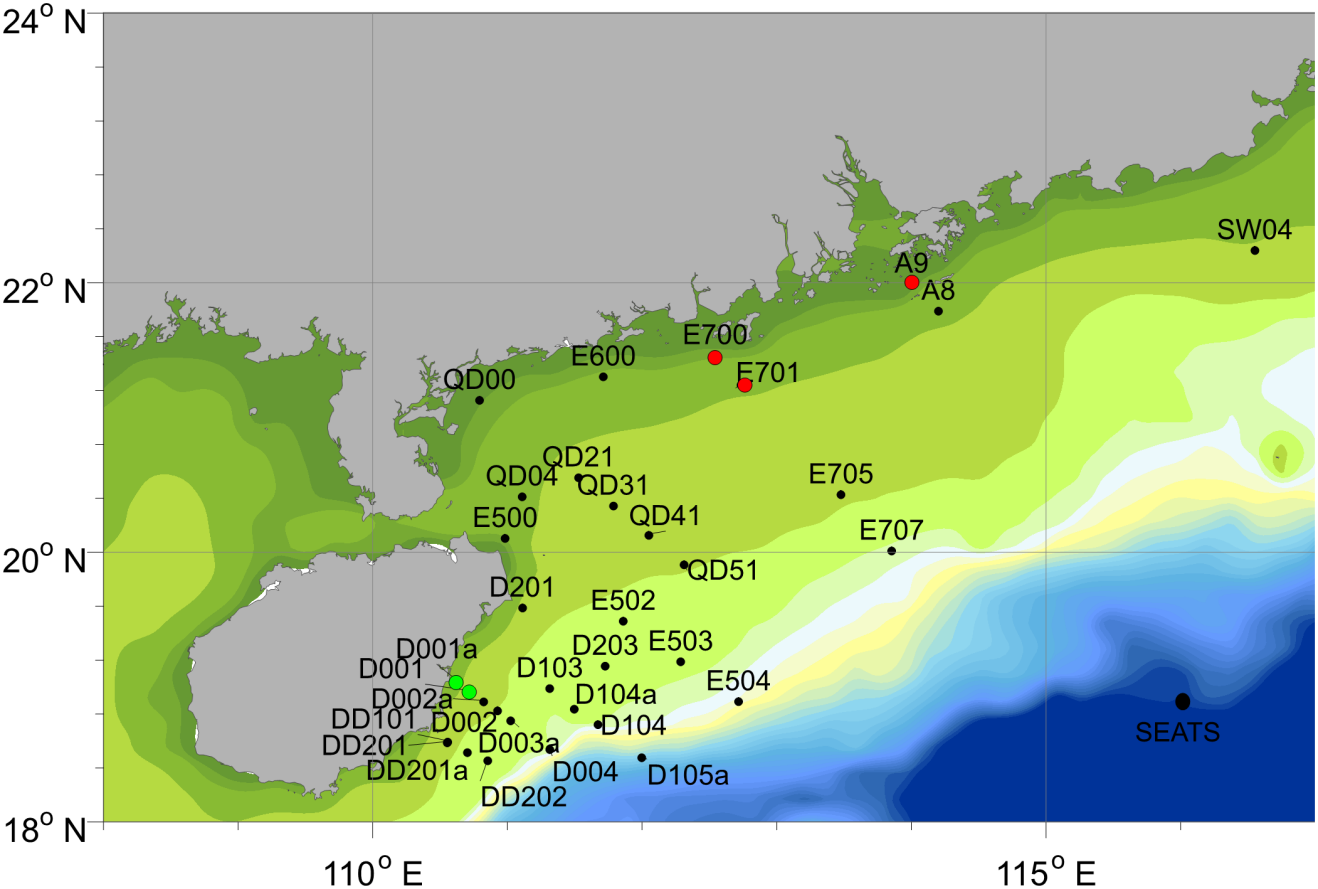
Experimental stations in the northern South China Sea. Red and green symbols represent the stations in river plume and upwelling area, respectively.

Our experiments were conducted at a total of 35 stations during a summer cruise (29 July to 23 August 2012) in the northern South China Sea (Fig 1). Detailed information of stations is given in Table 1. At each station, the In-Situ FIRe (Fluorescence Induction and Relaxation, Satlantic, Halifax, NS Canada) equipped with a 100 m cable was vertically released into the sea, with an approximate speed of 1.5 m s^-1^ (note, we released the 100 m cable downward but the real depth at each station was different due to hydrological conditions and locations) (Table 1). Water samples flowed through the optical head, which contains the pump/probe light emitting diodes (LEDs), reference photodiodes, fluorescence detection optics and pressure sensor. Duration of each measurement was 1.508 × 10^4^ μs. The interval between every two measurements was 4 seconds. Chl *a* fluorescence parameters [18], such as maximum photochemical efficiency of PSII in darkness (Fv/Fm) or effective photochemical efficiency of PSII under ambient light (Fq’/Fm’), quantum efficiency of photosynthetic electron transport (that reflects the effectiveness of photosynthetic apparatus in converting light energy into chemical reductant) and functional absorption cross section of PSII in darkness (σ_PSII_) or under ambient light (σ_PSII_’) (A^2^ quanta^-1^, ability of the photosynthetic apparatus to harvest light from the environment), were determined based on a single saturating turn-over flash (80 μs, 5 × 10^4^ μmol photons m^−2^ s^−1^). Fluorescence parameters were calibrated with blank measurements using filtered (0.2 μm) seawater from investigated area.

**Table 1:** Summary information of study stations during the cruise.

Station	Lat (°N)	Lon (°E)	Date (Local Time)	Measuring Time	Measuring Depth (m)	Surface PAR (μmol photons m^−2^ s^−1^)
**SW04**	22.2493	116.5520	30-July	11:00	20	400
**A9**	22.0011	114.0015	31-July	8:00	33	205
**A8**	21.7993	114.2014	02-Aug	11:03	42	684
**SEATS**	17.9963	115.9621	04-Aug	10:45	69	523
**E707**	20.0197	113.8537	08-Aug	15:20	28	1463
**E705**	20.4374	113.4773	08-Aug	21:00	37	0
**E701**	21.2511	112.7349	09-Aug	8:00	65	43
**E700**	21.4701	112.5429	09-Aug	11:12	19	2353
**QD00**	21.1375	110.7927	09-Aug	22:30	18	0
**E600**	21.3123	111.7120	09-Aug	16:40	28	1073
**QD04**	20.4218	111.1095	10-Aug	8:37	15	217
**QD21**	20.5631	111.5285	10-Aug	13:42	60	1244
**QD31**	20.3534	111.7877	10-Aug	17:02	50	1227
**QD41**	20.1358	112.0503	10-Aug	19:46	58	0
**QD51**	19.9172	112.3120	10-Aug	23:19	68	0
**E504**	18.9020	112.7167	11-Aug	12:35	40	286
**E503**	19.1978	112.2867	11-Aug	16:47	53	691
**E502**	19.4986	111.8600	11-Aug	22:24	70	0
**E500**	20.1130	110.9826	12-Aug	8:09	36	121
**D201**	19.5968	111.1125	12-Aug	14:03	53	552
**D203**	19.1652	111.7259	12-Aug	22:00	70	0
**D105a**	18.4857	111.9974	13-Aug	9:47	72	695
**D104**	18.7312	111.6724	13-Aug	15:10	72	595
**D104a**	18.8458	111.4964	13-Aug	19:08	80	2
**D103**	18.9988	111.3138	13-Aug	22:04	75	0
**D001a**	19.0502	110.6261	14-Aug	9:54	39	434
**D001**	18.9740	110.7166	14-Aug	12:14	72	243
**D002a**	18.9002	110.8235	15-Aug	15:33	68	81
**D002**	18.8332	110.9266	15-Aug	17:05	88	508
**D003a**	18.7595	111.0239	15-Aug	19:00	94	6
**D004**	18.5462	111.3147	15-Aug	23:52	93	0
**DD202**	18.4633	110.8537	16-Aug	8:19	90	131
**DD201a**	18.5235	110.7027	16-Aug	10:37	82	562
**DD201**	18.5965	110.5529	16-Aug	12:30	70	285
**DD101**	18.6018	110.5563	17-Aug	0:02	32	189

*In-situ* light intensities were measured in parallel by a photosynthetically active radiation (PAR) sensor (Satlantic, Halifax, NS Canada) attached to the top of the instrument. Seawater temperature, salinity and pressure were also measured with a CTD system (Seabird 911).

### 2.2 Ethics statement

There are no specific permits required for the described sampling because collections did not involve endangered species and did not occur within a designated marine protected area, private reserve or park.

### 2.3 Data analyses

Liner fitting analysis was used to test the relationships between photosynthetic parameters of functional absorption cross section (σ_PSII_ or σ_PSII_’), or photochemical efficiency (Fv/Fm or Fq’/Fm’) and depth.

## 3. Results

### 3.1 Data overview of the photosynthetic parameters

**Fig 2:**
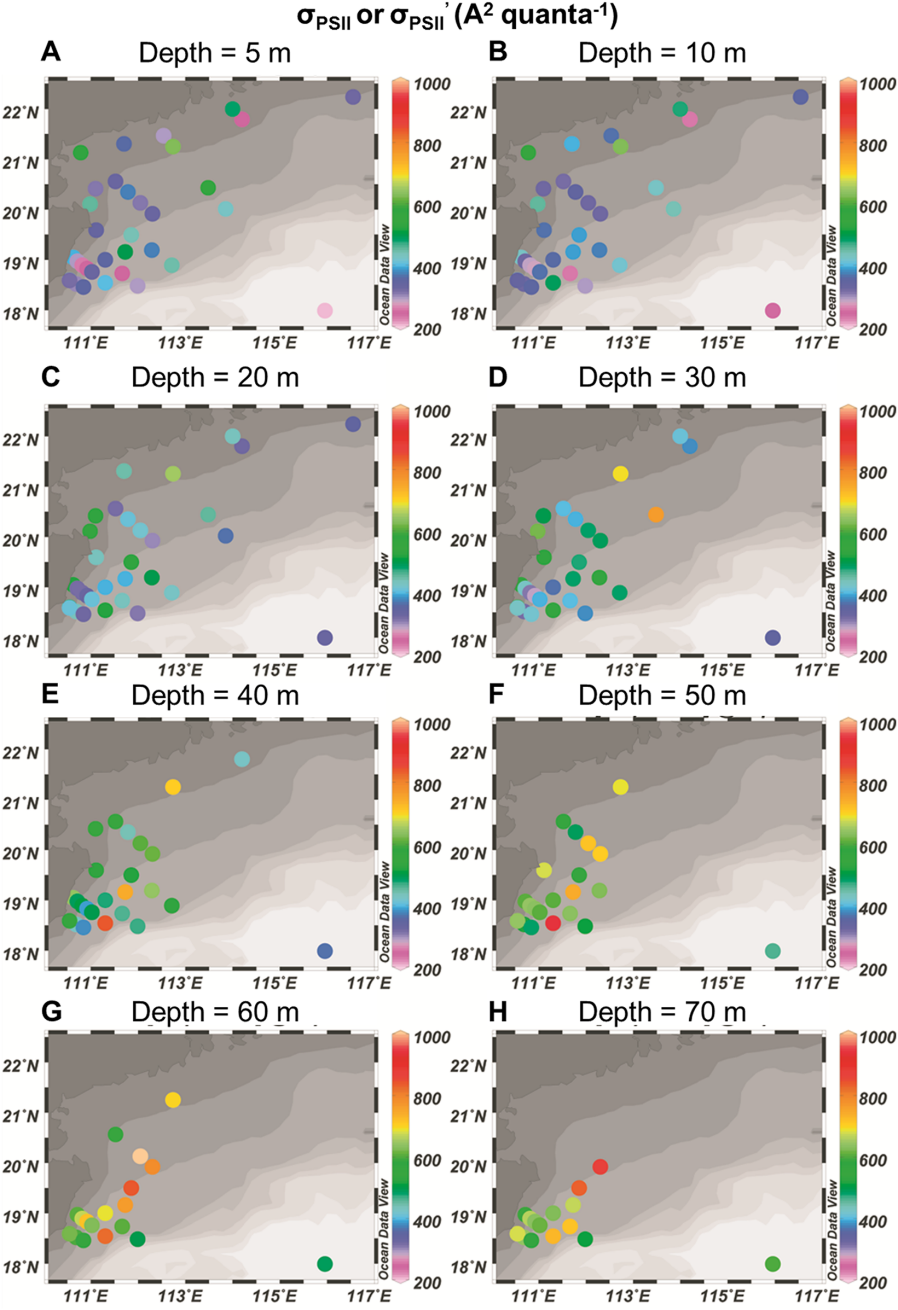
Horizontal and vertical distributions of functional absorption cross section of photosystem II (PSII) in darkness (σ_PSII_) or under ambient light (σ_PSII_’) (A^2^ quanta^-1^) at 5 (A), 10 (B), 20 (C), 30 (D), 40 (E), 50 (F), 60 (G) and 70 m (H) depth, respectively.

**Fig 3:**
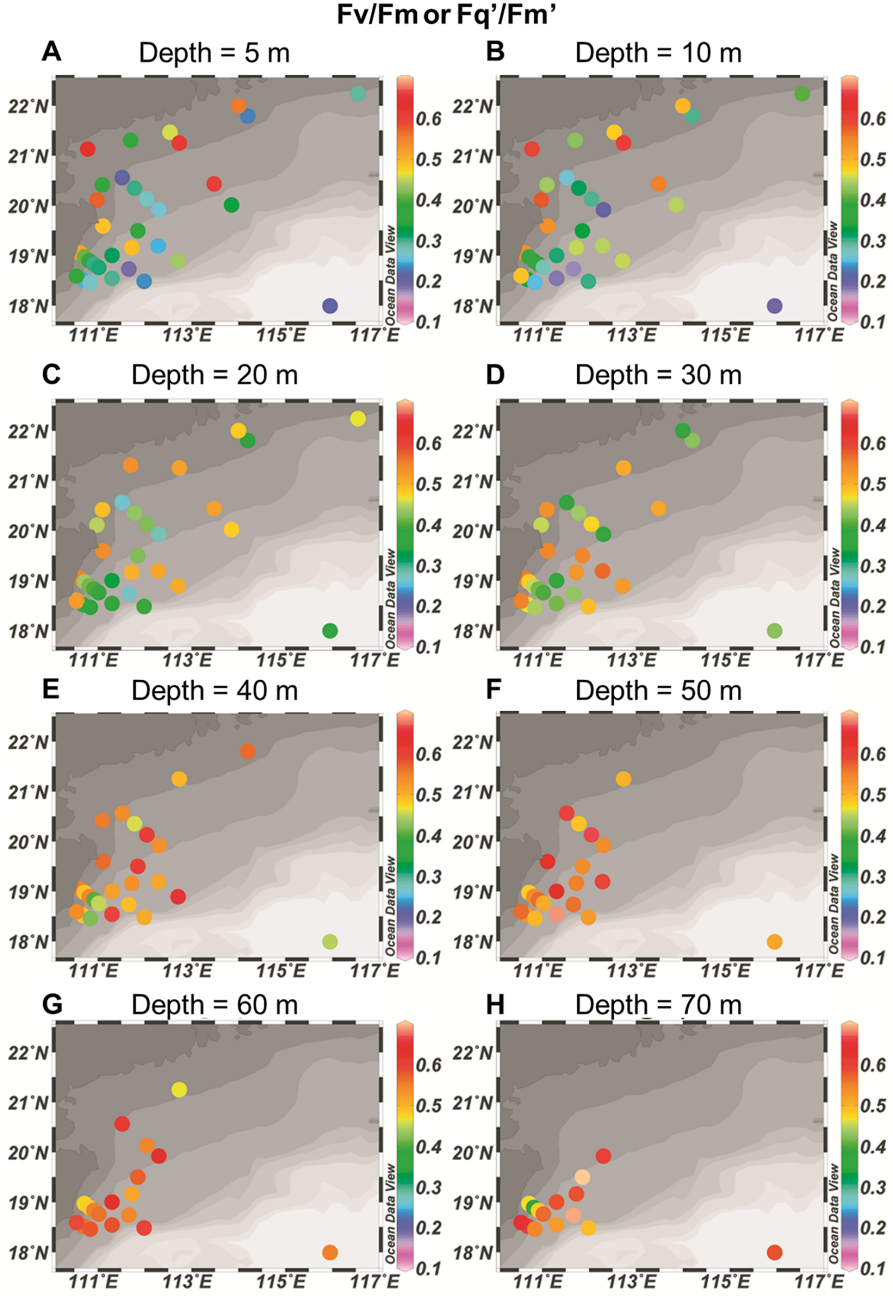
Horizontal and vertical distributions of maximum (Fv/Fm, measured in darkness) or effective (Fq’/Fm’, measured under ambient light) photochemical efficiency of photosystem II (PSII) at 5 (A), 10 (B), 20 (C), 30 (D), 40 (E), 50 (F), 60 (G) and 70 m (H) depth, respectively.

The functional absorption cross section of PSII (**σ**_PSII_ in darkness or σ_PSII_’ under ambient light, A^2^ quanta^-1^) increased with depths at all the stations regardless of the measuring time (12 stations at night and 23 stations at daytime, Table 1). The functional absorption cross section of PSII ranged from 218 to 606 A^2^ quanta^-1^ with an average value of 357 A^2^ quanta^-1^ in the surface water (Fig 2A) and with increased depths, it ranged from 297 to 1000 A^2^ quanta^-1^ (average = 526 A^2^ quanta^-1^) at the bottom (the averaged bottom values were obtained across the 5 m range from the maximum measuring depth up to 5 m the maximum depth) (Fig 2F). The highest surface functional absorption cross section of PSII was 606 A^2^ quanta^-1^, recorded at station E701, while the lowest surface value of 218 A^2^ quanta^-1^, was recorded at station SEATS (Fig 2A). The maximum (Fv/Fm) or effective photochemical efficiency of PSII (Fq’/Fm’) increased with depths at most stations (Fig 3). Fv/Fm or Fq’/Fm’ ranged from 0.191 to 0.658 with an average value of 0.377 in the surface water (Fig 3A), and 0.307 to 0.781 (average = 0.538) at the bottom (Fig 3F). Highest surface photochemical efficiency reached up to 0.658 at station E701 (Fig 3A). Station D105a had the lowest surface Fq’/Fm’ of 0.191 (Fig 3A).

### 3.2 Photosynthetic parameters in Pearl River plume areas

**Fig 4:**
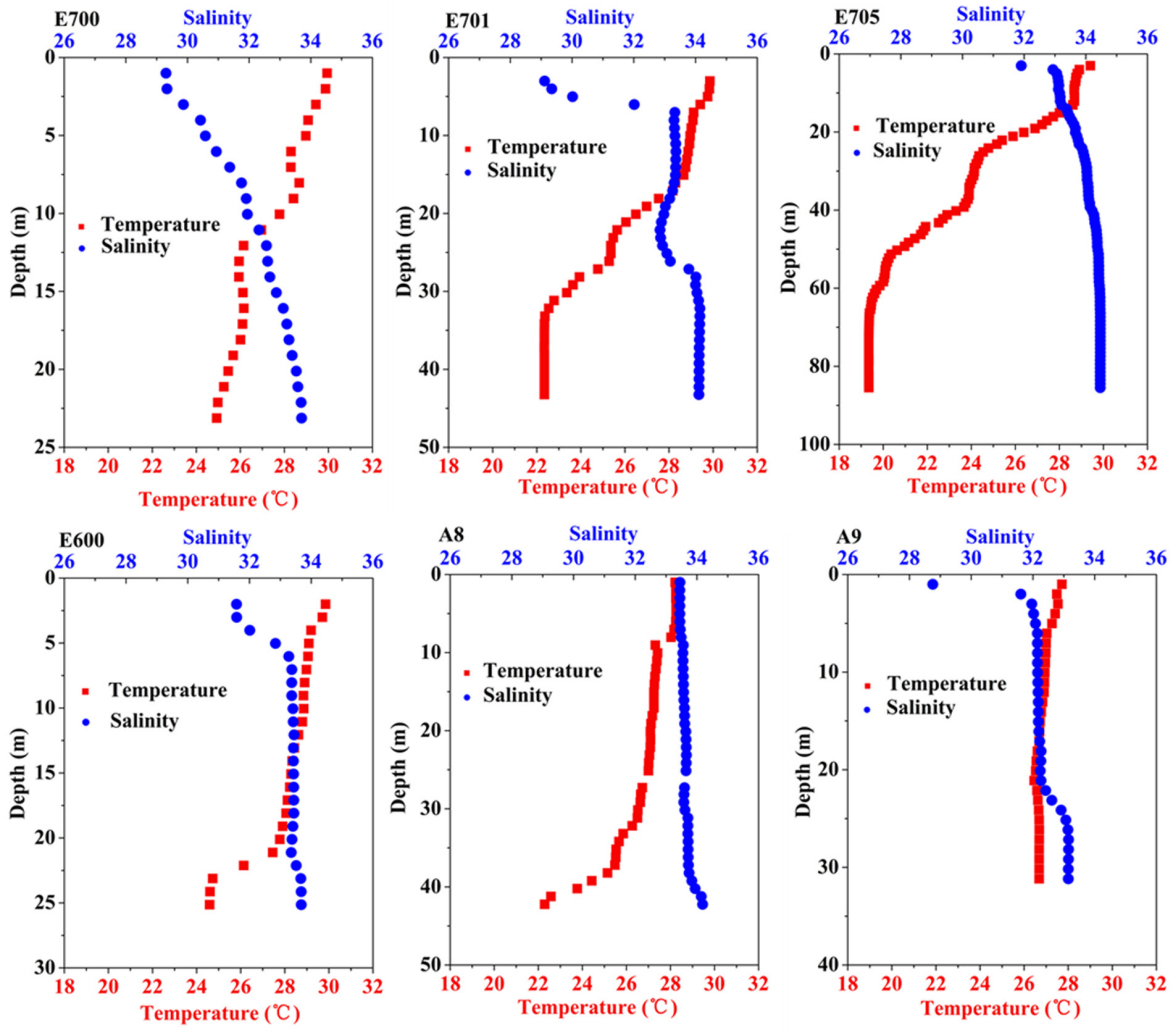
Vertical profiles of salinity (PSU, blue) and temperature (°C, red) of station E700 (A), E701 (B), E705 (C), E600 (D), A8 (E) and A9 (F).

**Fig 5:**
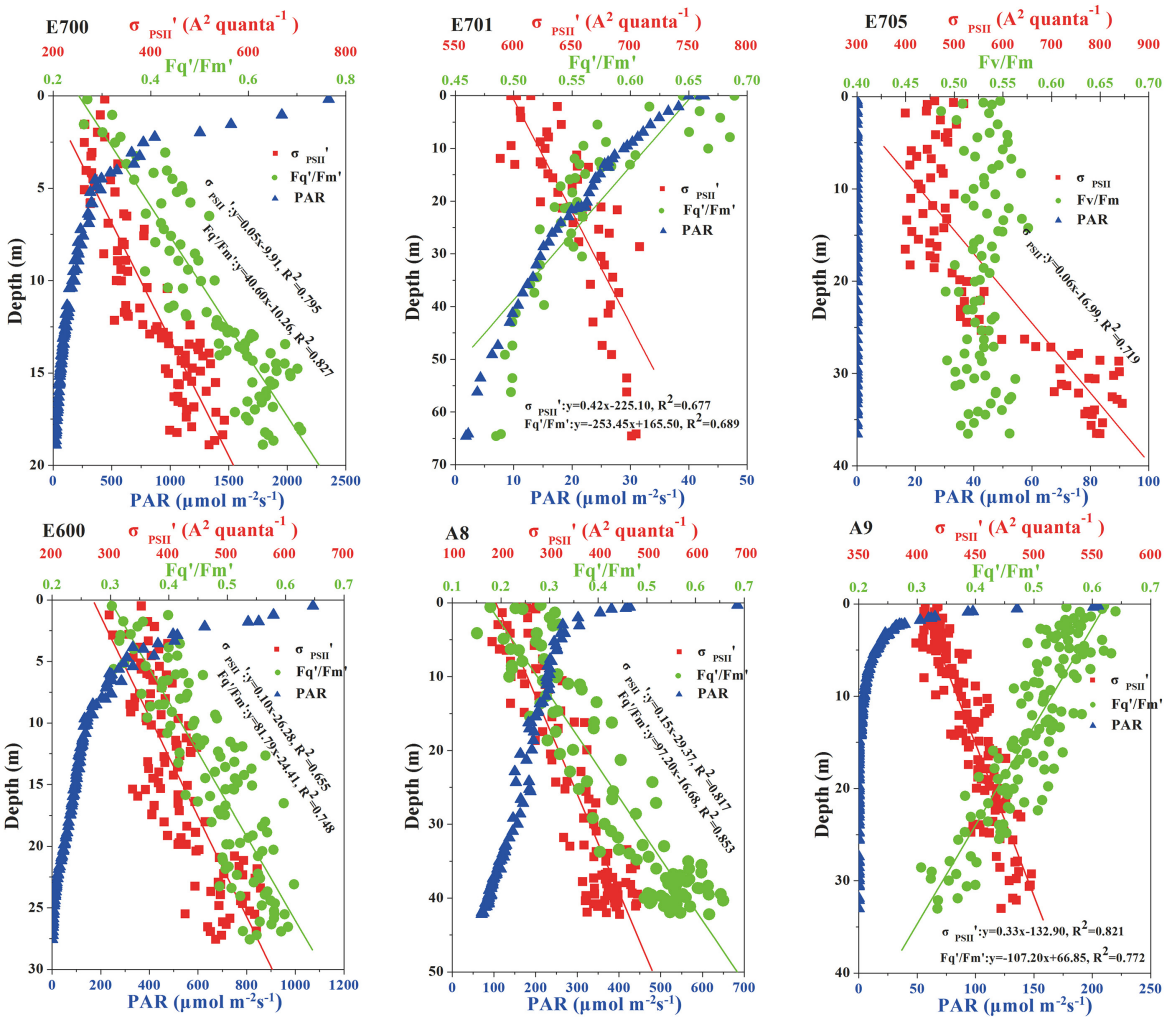
Vertical profiles of photosynthetic parameters of functional absorption cross section of photosystem II (PSII) in darkness (σ_PSII_) or under ambient light (σ_PSII_’) (A^2^ quanta^-1^) (red square) and maximum (Fv/Fm, measured in darkness) or effective (Fq’/Fm’, measured under ambient light) photochemical efficiency of photosystem II (PSII) (green circle) and photosynthetically active radiation (PAR) (blue triangle) irradiance of station E700 (A), E701 (B), E705 (C), E600 (D), A8 (E) and A9 (F), respectively. The solid lines denote the regression curve between functional absorption cross section (σ_PSII_ or σ_PSII_’) (red), or photochemical efficiency (Fv/Fm or Fq’/Fm’) (green) and depth.

Three stations of A9, E700, and E701 were affected by the Pearl River plume during our cruise, as reflected by low salinity (~29 psu), (Fig 4) and high nutrient concentrations [*Dai et al*., unpublished data] at the surface. In order to investigate the photochemical changes of phytoplankton induced by river plume, we plotted the vertical profiles of station A9, E700, and E701 in the Pearl River plume plus two stations of A8, E705, and E600 near the Pearl River Plume areas (Fig 5). Vertical profiles of functional absorption cross section of PSII for the stations A8, A9, E700, E701, E705 and E600 showed that they increased with depth, with a slope of 2.38–20 m^-1^, regardless of the freshwater discharges. At the surface, σ_PSII_ or σ_PSII_’ was 182–637 A^2^ quanta^-1^, at the bottom it was 375–705 A^2^ quanta^-1^. In contrast, the photochemical efficiency only increased with the depth in the stations of E700, E600 and A8 (slope ranges from 0.010 to 0.025 m^-1^) (Fig 5A, 5D and 5E), while at the stations of E701 and A9, it decreased with the depth with a slope of -0.004 and -0.009 m^-1^, respectively (Fig 5B and 5F). Interestingly, at station E705, there was no obvious change of photochemical efficiency from the surface (0.51–0.57) to the bottom (0.49–0.50) (Fig 5C). The highest value of photochemical efficiency in the surface was 0.69 at station E701 (Fig 5B), while the lowest value was 0.15 at station A8 (Fig 5E). At the bottom, highest photochemical efficiency of 0.71 was found in station E700 (Fig 5A), whilst station A9 had the lowest photochemical efficiency of 0.31 (Fig 5F).

### 3.3 Photosynthetic parameters in upwelling regions

**Fig 6:**
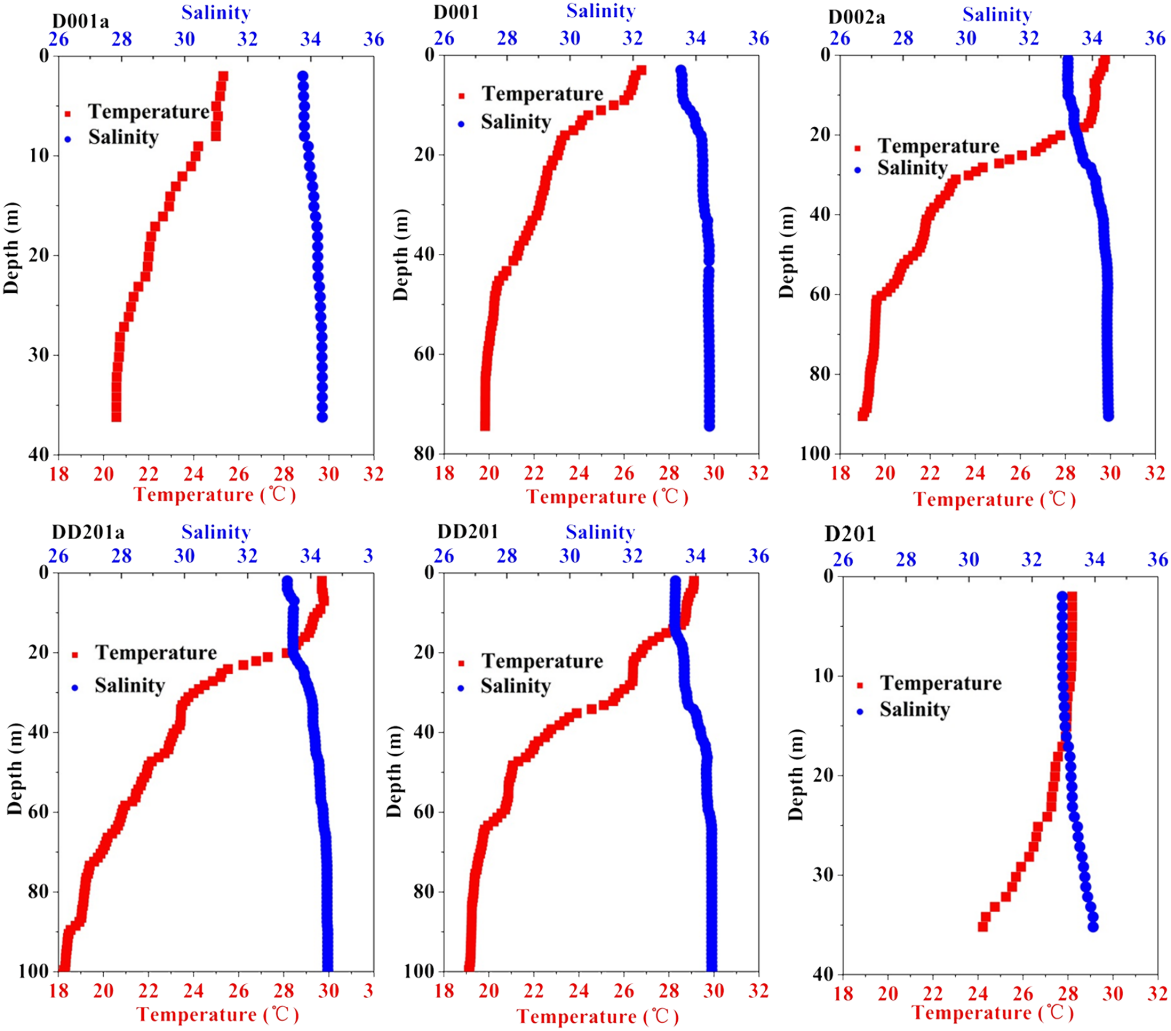
Vertical profiles of salinity (PSU, blue) and temperature (°C, red) of station D001a (A), D001 (B), D002a (C), DD201a (D), DD201 (E) and D201 (F).

**Fig 7:**
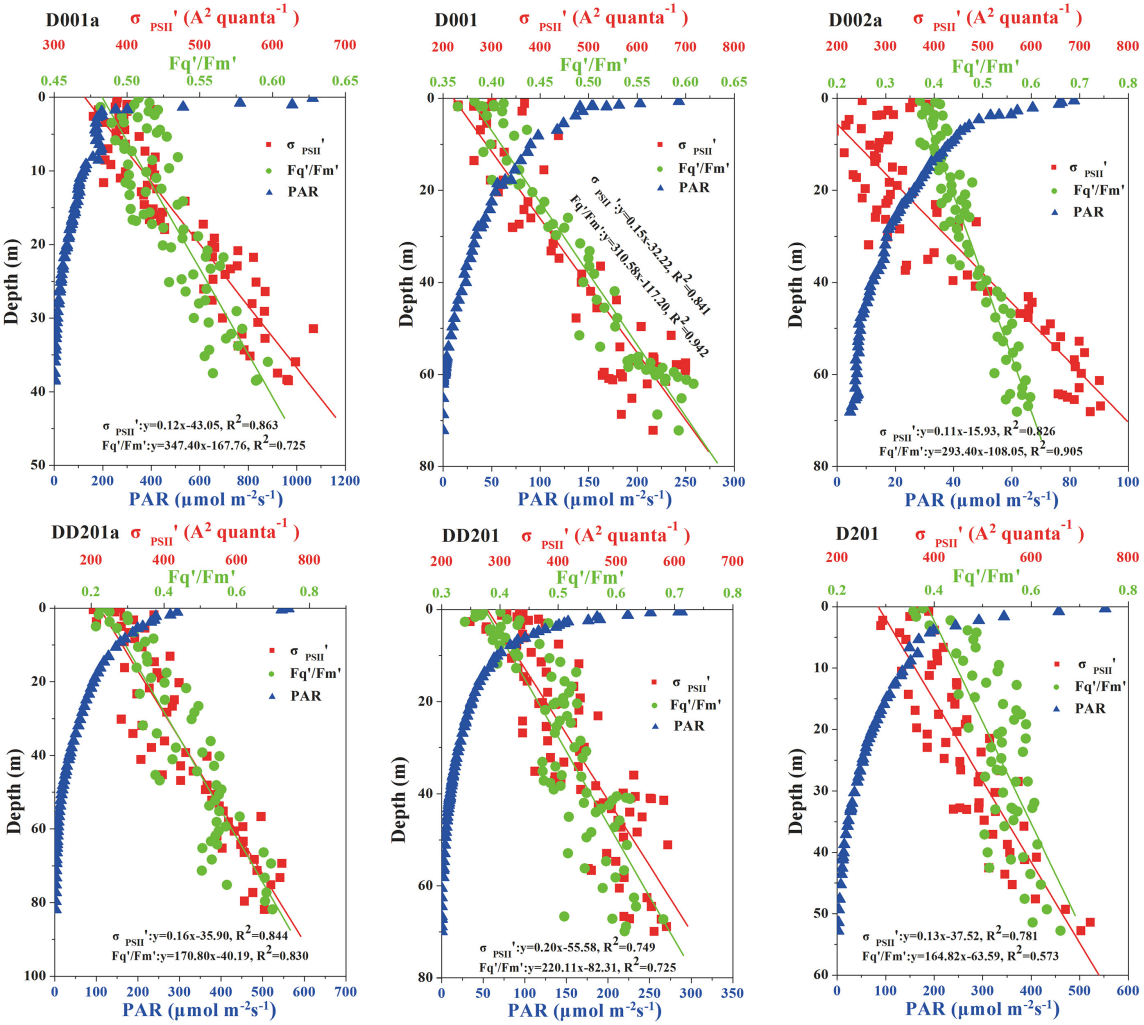
Vertical profiles of photosynthetic parameters of functional absorption cross section of photosystem II (PSII) under ambient light (σ_PSII_’) (A^2^ quanta^-1^) (red square) and effective (Fq’/Fm’, measured under ambient light) photochemical efficiency of photosystem II (PSII) (green circle) and photosynthetically active radiation (PAR) (blue triangle) irradiance of station D001a (A), D001 (B), D002a (C), DD1201a (D), DD201 (E) and D201 (F). The solid lines denote the regression curve between functional absorption cross section (σ_PSII_’) (red), or effective photochemical efficiency (Fq’/Fm’) (green) and depth.

The stations in the upwelling regions featured with low sea surface temperature (SST) and high salinity (D001a and D001, Fig 6A and 6B). In order to examine the effects the upwelling on the photosynthetic performance of phytoplankton, we plotted the vertical profiles of station D001a and D001 in upwelling regions, and the other four stations of D002a, DD201a, DD201 and D201 near the upwelling regions as comparisons as shown in Fig 7. In general, both the Fq’/Fm’ and σ_PSII_’ (they were all measured at daytime under ambient light) increased with depth at all these 6 stations (Fig 7). The slope was 5 to 9.09 m^-1^ with σ_PSII_’ and 0.003 to 0.006 m^-1^ with Fq’/Fm’. At the surface, σ_PSII_’ ranged from 162 to 462 A^2^ quanta^-1^, while at the bottom it was 463–743 A^2^ quanta^-1^. The Fq’/Fm’ ranged from 0.13 to 0.57 in the surface waters and 0.39 to 0.72 at the bottom (Fig 7).

## 4. Discussion

**Fig 8:**
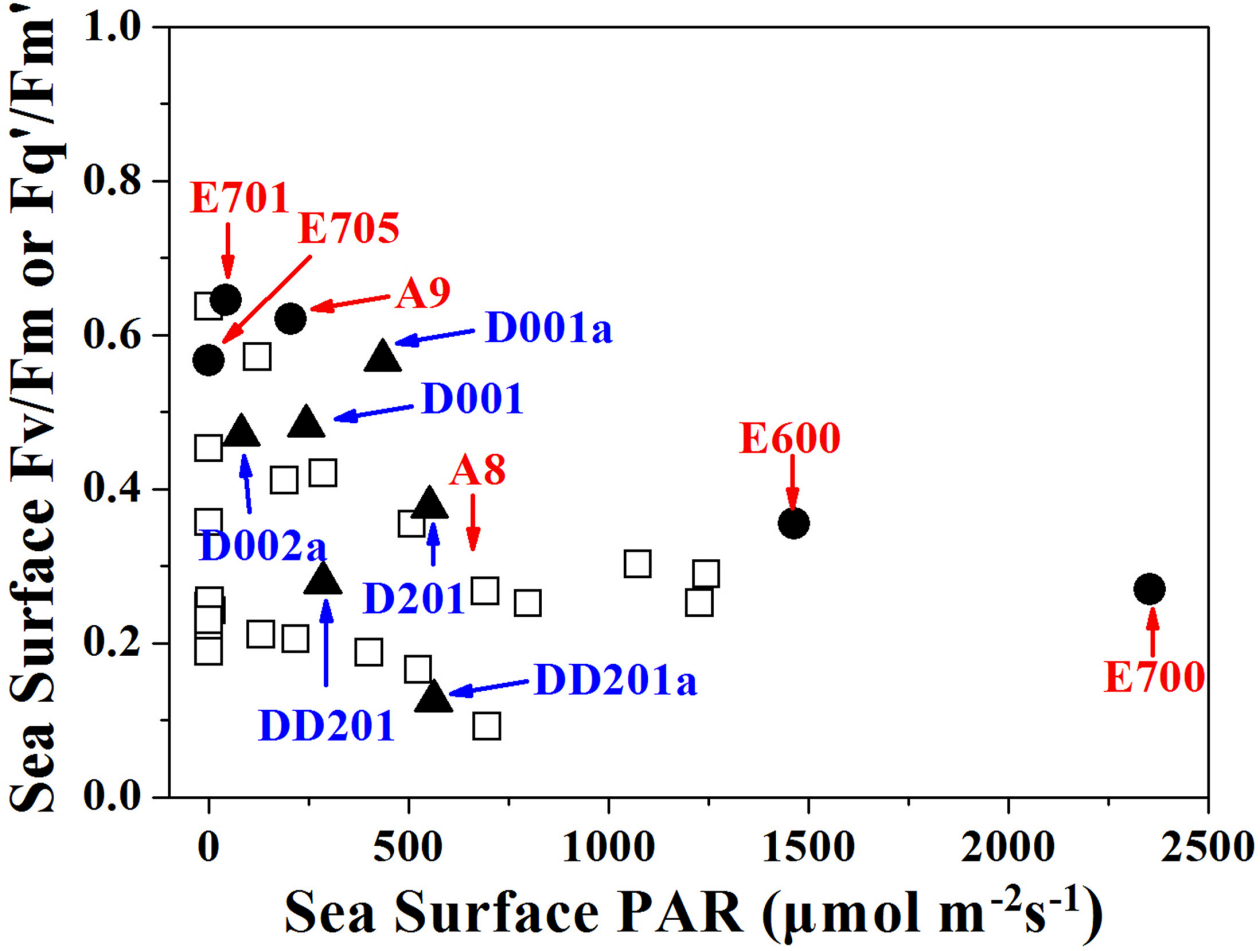
The sea surface yield in all the studied stations as a function of sea surface photosynthetically active radiation (PAR) levels (μmol photons m^−2^ s^−1^). Solid circles and triangles represent the stations in river plume and upwelling areas, respectively. The letters and numbers indicate stations.

In the stations that covered 4.0 × 10^5^ km^2^, we observed that the maximum or effective photochemical efficiency (Fv/Fm or Fq’/Fm’ in present study), which reflects photosynthetic performance, negatively correlated well with incident light levels either at different depths or at the surface during different measuring times when sunlight fluctuated (Fig 8). Surface phytoplankton cells with high light exposures often suffer from photodamages from UV radiation in addition to excessive PAR and exhibit low photochemical efficiency of PSII [19]. For most of the stations that were investigated during noon or high-light exposures, the photochemical efficiency of PSII was low in the surface waters (Figs 3 and 5).

Many phytoplankton species modulate the effective absorption cross section of PSII to enable acclimation and then adaptation over a wide range of irradiance [20, 21]. Effective absorption cross section of PSII (σ_PSII_’) can be controlled by various environmental factors such as cell size [21, 3], taxonomic composition [3], nutrient status [3] and ultraviolet radiation [22, 23]. Although phytoplankton community structure [24–26], nutrient status [27] differed in all the studied stations in present study, the σ_PSII_’ all increased with the depth (Figs 2, 5 and 7), reflecting an increased light use efficiency at lower irradiance in deeper waters. Such a phenomenon could be photoadaptation strategy, reflecting a genotypic response to vertical irradiance changes [28]. However, the estimated PSII contents (expressed as [RCII] in nmol m^-3^, S1 Text, S1 Fig) did not show obvious changes within the depth, indicating that phytoplankton adapt to the wide range of irradiance by modulating the absorption cross section of PSII rather than changing the contents of PSII (S1 Fig). These results are in a good agreement with some of the previous studies which showed that the photosystem II function varies independently from their contents [29].

It is known that riverine inputs of material are the primary source of nutrients sustaining shelf ecosystems, and this is particularly true in large river-shelf ecosystems where river discharge dominates the shelf nutrient dynamics and thus biological productivities [30]. Therefore, the physico-chemical and biological changes induced by freshwater discharges will affect the photosynthetic performances of phytoplankton in these areas. In the present study, the photochemical efficiency at stations (A9 and E701) in the Pearl River plume decreased with depth, which was opposite to the change pattern found at stations (A8 and E600) near or at the edge of the plume (Fig 5). In river plumes, phytoplankton cells with high availability of nutrients can tolerate high light or UV radiation levels, so that their photosynthetic machinery suffers from less damages [31], consequently, their photochemical efficiency could sustain high levels even during high light exposures (e.g. A9 and E701). At the station E705, representative of the marginal area of Pearl River plume, suppression of photochemical efficiency towards the surface due to high light intensity at day time was probably fully or partially offset by the positive effects of increased nutrient availability or dark repairing (measured at 21:00 at night), so that no obvious changes of photochemical efficiency from the surface to the bottom were found (Fig 5). Furthermore, the photochemical efficiency at station E700 increased with depth even it located in the area of Pearl River plume. This might be due to the extremely strong solar radiation (up to 2500 μmol photons m^-2^ s^-1^) on the seawater surface, which overpowered the enhancement brought along by sufficient nutrient.

Upwelling brings nutrient-replete, high *p*CO_2_ waters to the surface, which can affect the photosynthetic performance of phytoplankton. Nevertheless, in the present study, the photochemical efficiency (termed as Fv/Fm or Fq’/Fm’) and functional absorption cross section of PSII (termed as σ_PSII_ or σ_PSII_’) of phytoplankton at the stations in upwelling areas (D001a, D001) showed similar trends with those outside of it with little influence of the upwelling event (Fig 7). Relative higher nutrients in the surface waters were predicated to increase the photochemical efficiency as we discussed above, at the same time, elevated *p*CO_2_ [Dai *et al*., unpublished data] was suggested to enhance phytoplankton photochemical efficiency as well [32], however, the lower temperature in the upwelled seawater could decrease the photochemical efficiency since phytoplankton grown at lower temperature usually has limited linear electron transport due to low ribulose-1, 5-bisphosphate carboxylase/oxygenase (RUBISCO) activity and slower metabolic repair activity [33]. Likewise, the positive correlation between photochemical efficiency of PSII and temperature was reported in the sub-Antarctic and Polar Frontal Zone [34]. Therefore, in the upwelling areas, it appeared that the nutrients-stimulation and low SST-suppression could be neutralized so that no obvious discrepancy was found within and outside of the upwelling area.

It has been demonstrated that primary productivity was very high in the costal river plume [35], and upwelling areas [36, 37]. Higher availability of nutrients in these areas was considered to be responsible for the high primary productivity [35]. In the present study, higher phytoplankton photochemical efficiency and estimated chlorophyll *a* specific photosynthetic electron transport rate through PSII (ETR_PSII_) (expressed as mol e^-1^ [mol chl *a*]^-1^ s^-1^, S1 Text, S2 Fig) were observed in these stations (e.g. E701, A9, D001a) (Figs 5, 7 and S2 Fig), which coincides with the high primary productivity reported previously [35, 37]. Apparently, *in-situ* chlorophyll fluorescence monitoring technique could be applied as a potential proxy to probe physiological performance as well as to estimate primary productivity.

Environmental forcing generates selective pressures on the genotypes present within an ecosystem, resulting in changes in phytoplankton community structure. It can also drive physiological (phenotypic) responses that may ameliorate or exacerbate these selective pressures. Here, we observed marked changes in photochemical efficiency and effective absorption cross section of PSII of phytoplankton in NSCS. While the extent to which these physiological parameters were directly influenced by resource limitation or indirectly reflected environmental forcing through shifts in community structure, is unclear, our data demonstrated that the functional absorption cross section of PSII was a genotypic response of the phytoplankton to irradiance (niche partition) in the NSCS, while the photochemical efficiency of PSII was more a photoacclimation strategy for phytoplankton and more flexible with changes in physical and chemical environmental changes.

## Supporting Information

S1 Dataset The whole dataset of this paper. (XLSX)

S1 Fig Horizontal and vertical distributions of concentration of functional PS II reaction centers ([RCII], ×100 nmol m^-3^) at 5 (A), 10 (B), 20 (C), 30 (D), 40 (E), 50 (F), 60 (G) and 70 m (H) depth, respectively. (TIF)

S2 Fig Horizontal and vertical distributions of chlorophyll *a* specific ETR (mol e^-1^ [mol chl *a*]^-1^ s^-1^) at 5 (A), 10 (B), 20 (C), 30 (D), 40 (E), 50 (F), 60 (G) and 70 m (H) depth, respectively. (TIF)

S1 Text Estimation of the chlorophyll *a* specific ETR (ETR_PSII_) and the concentration of functional PS II reaction centers ([RCII]). (DOCX)
