# Supplementary figures and images for: Contrasting Photophysiological Characteristics of Phytoplankton Assemblages in the Northern South China Sea
Source: PLoS One. 2016 May 19;11(5):e0153555. doi: 10.1371/journal.pone.0153555 (PMC4873168; doi:10.1371/journal.pone.0153555)

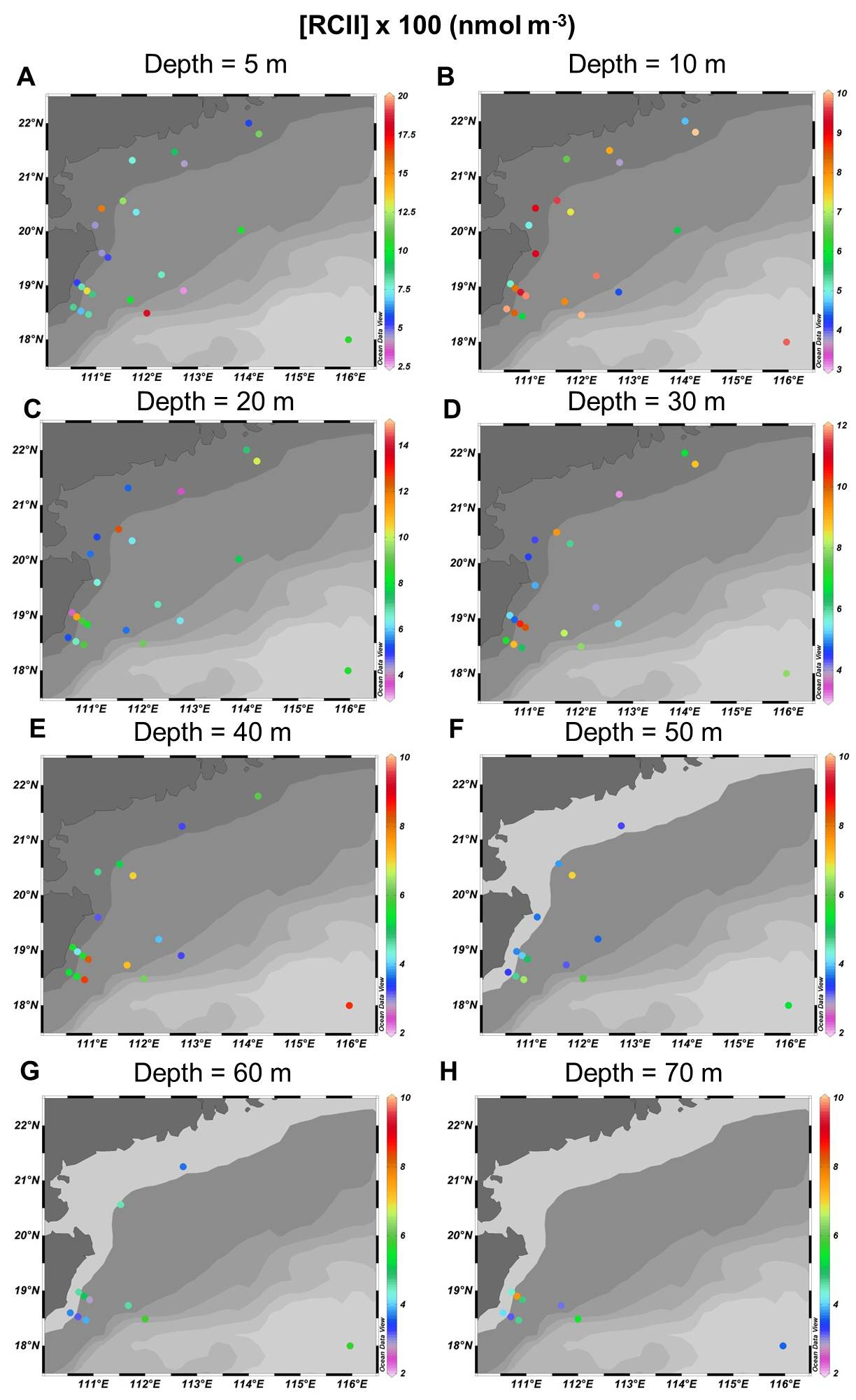

Supplement: S1 Fig — (TIF) [file pone.0153555.s002.tif]

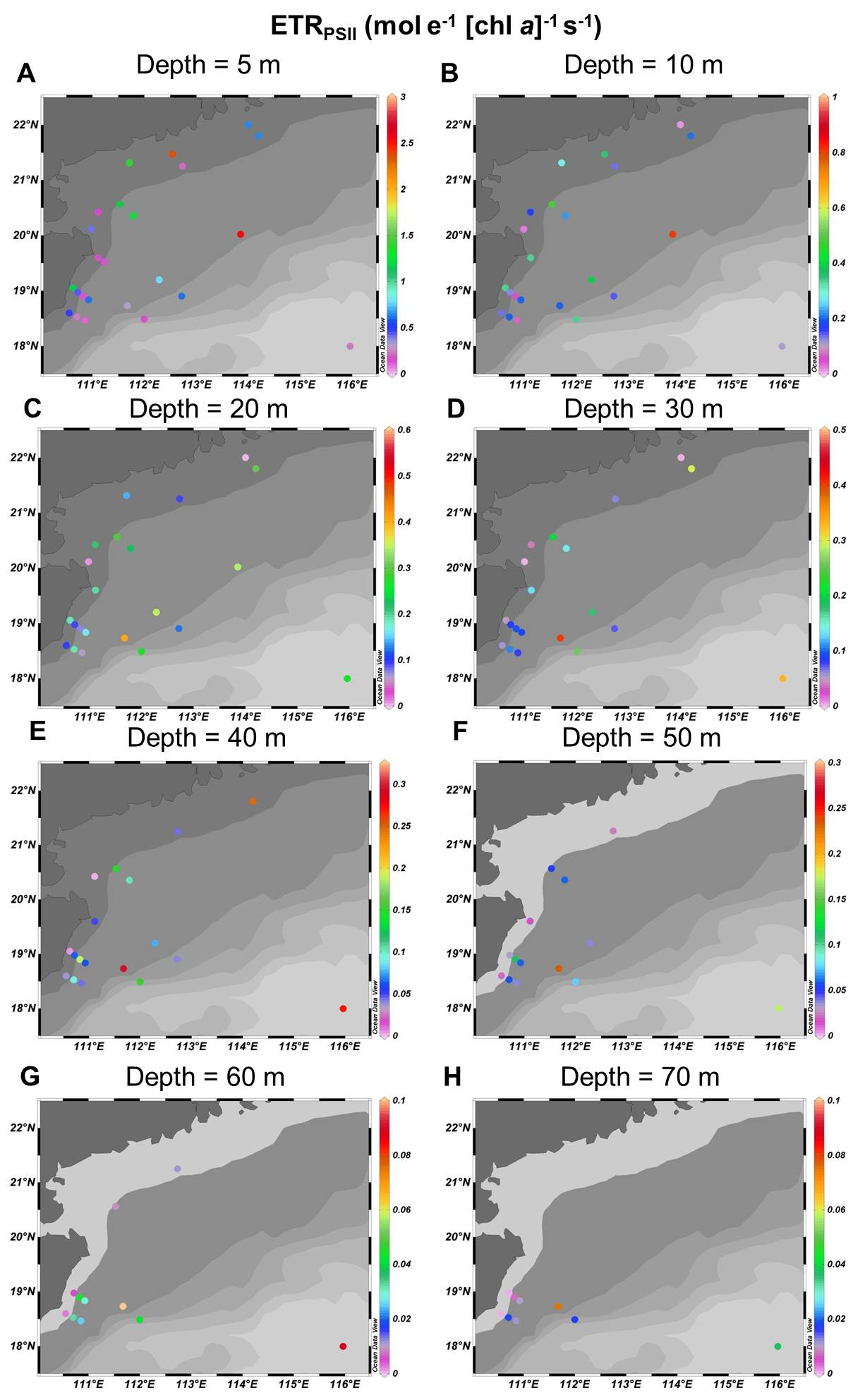

Supplement: S2 Fig — (TIF) [file pone.0153555.s003.tif]
