# Supplementary material for: Contrasting Photophysiological Characteristics of Phytoplankton Assemblages in the Northern South China Sea
Source: PLoS One. 2016 May 19;11(5):e0153555. doi: 10.1371/journal.pone.0153555 (PMC4873168; doi:10.1371/journal.pone.0153555)
Supplement: S1 Text — (DOCX) [file pone.0153555.s004.docx]

**Estimation of the chlorophyll *a* specific ETR (ETR_PSII_)**

We estimated the chlorophyll *a* specific ETR (mol e^-1^ [mol chl *a*]^-1^ s^-1^) by following equation (Suggett et al. 2011, Chapter 6 in *Chlorophyll a fluorescence in aquatic sciences: methods and applications*, pp. 103–127):

ETR_PSII_ = σ_PSII_’ × Φ_PSII_ × n_PSII_× E (1)

where σ_PSII_’ (A^2^ quanta^-1^) is the functional absorption cross section of PSII under ambient light, Φ_PSII_ (dimensionless) is the PSII photochemical efficiency under ambient light, E (μmol photons m^−2^ s^−1^) is the ambient light density and n_PSII_ is a measure of the ratio of functional PSII reaction center (RCII) concentration to total chlorophyll *a* present.

n_PSII_ is defined as,

n_PSII_ = RCII/Chl *a*  (2)

Here we used 0.002 as a “typical” value that has been frequently employed as proposed by Kolber and Falkowski (Limnol Oceanogr. 1993; 38: 1646-1665). The readers should be aware that we only estimated the ETR_PSII_ that measured at daytime, because E in equation 1 is nearly 0 at the measuring time of night.

**Estimation of concentration of functional PS II reaction centers ([RCII])**

We estimated concentration of functional PS II reaction centers ([RCII], m^-3^) by following the approach proposed in Oxborough et al. 2012 (Limnol. Oceanogr.: Methods 10, 2012, 142–154):

[RCII] = $\frac{K_{R}}{E}$ × $\frac{F_{0}'}{\sigma PSII'}$ (3)

where K_R_ (photons m^−3^ s^−1^) is the instrument specific constant (2.59 × 10^21^ as employed by Oxborough et al. 2012, Limnol. Oceanogr.: Methods 10, 2012, 142–154 ), E (photons m^−2^ s^−1^) is the measuring beam intensity (5 × 10^4^ in the present study), F_0_’ is the fluorescence at zeroth flashlet of an single turnover flash measurement when *C* = 0 (*C* is the fraction of RCII in the closed state) under ambient light, and σ_PSII_’ (A^2^ quanta^-1^) is the functional absorption cross section of PSII under ambient light.
